# Hypomethylation‐activated cancer‐testis gene SPANXC promotes cell metastasis in lung adenocarcinoma

**DOI:** 10.1111/jcmm.14532

**Published:** 2019-09-04

**Authors:** Xuewei Wang, Sihan Ju, Yao Chen, Qufei Qian, Caiwang Yan, Shuaizhou Chen, Yuting Chang, Yide Xu, Zijian Ma, Chang Zhang, Na Qin, Yayun Gu, Cheng Wang, Erbao Zhang, Zhibin Hu

**Affiliations:** ^1^ Department of Epidemiology and Biostatistics, Center for Global Health, School of Public Health Nanjing Medical University Nanjing China; ^2^ State Key Laboratory of Reproductive Medicine Nanjing Medical University Nanjing China; ^3^ Jiangsu Key Lab of Cancer Biomarkers, Prevention and Treatment, Jiangsu Collaborative Innovation Center for Cancer Personalized Medicine Nanjing Medical University Nanjing China; ^4^ Department of Bioinformatics, School of Basic Medical Sciences Nanjing Medical University Nanjing China

**Keywords:** cancer‐testis, lung adenocarcinoma, metastasis, ROCK1, SPANXC

## Abstract

Many studies have shown that there were similarity between tumorigenesis and gametogenesis. Our previous work found that cancer‐testis (CT) genes could serve as a novel source of candidate of cancer. Here, by analysing The Cancer Genome Atlas (TCGA) database, we characterized a CT gene, SPANXC, which is expressed only in testis. The SPANXC was reactivated in lung adenocarcinoma (LUAD) tissues. And the expression of SPANXC was associated with prognosis of LUAD. We also found that the activation of SPANXC was due to the promoter hypomethylation of SPANXC. Moreover, SPANXC could modulate cell metastasis both in *vitro* and in *vivo*. Mechanistically, we found that SPANXC could bind to ROCK1, a metastasis‐related gene, and thus SPANXC may regulate cell metastasis partly through interaction with ROCK1 in LUAD. Together, our results demonstrated that the CT expression pattern of SPANXC served as a crucial role in metastasis of LUAD. And these data further corroborated the resemblance between processes of germ cell development and tumorigenesis, including migration and invasion.

## 1 INTRODUCTION

Lung cancer is the most common cancer type and the leading cause of cancer death.^1^ Lung adenocarcinoma (LUAD) is the most diagnosed histological subtype of non–small‐cell lung cancer (NSCLC).^2^ Even though rapid development of radiotherapy, chemotherapy and targeted therapy for NSCLC, the overall 5‐year survival rate is poor due to limited treatment options, and tumour recurrence and metastasis.^3^, ^4^, ^5^ There is no doubt that the insight of tumorigenesis could provide new and efficacious treatments for NSCLC.

Cancer‐testis (CT) genes are a group of genes which show a restrictive expression pattern in testis.^6^ Several known cancer‐testis antigens have contributed to spermatogenesis and carcinogenesis.^6^ CT genes were frequently activated in multiple different tumour types.^7^ For example, CT genes MAGE‐A3 could serve as an independent prognostic marker for patients of LUAD.^8^ Our previous studies systematically defined CT genes in 19 cancer types and investigated their potential role in tumorigenesis.^9^, ^10^ We also found that CT long non‐coding RNA LIN28B‐AS1 could regulate biological function of LUAD cells by direct interaction to IGF2BP1 in LUAD.^11^

SPANXC is a member of the SPANX (sperm protein associated with the nucleus in the X chromosome) gene family.^12^, ^13^ SPANX gene family encodes differentially expressed testis‐specific proteins. These proteins were activated in prostate, lung and bladder cancers.^14^ SPANXC could promote breast cancer cell invasion by interacting with structural proteins lamin A/C.^15^ These studies indicated that CT gene SPANXC could exhibit a pivotal role in tumorigenesis. However, the role of SPANXC in LUAD had not yet been established.

Here, we present evidence that the elevated expression of CT gene SPANXC was associated with poor LUAD patient prognosis in TCGA LUAD samples. Further analysis found that promoter hypomethylation could lead to the activation of SPANXC in LUAD samples of TCGA. Moreover, SPANXC could modulate cell metastasis both in *vitro* and in *vivo*. Mechanistically, we found that SPANXC could directly interact with ROCK1 in LUAD cells. Together, our study firstly identified that SPANXC is an important CT gene in tumorigenesis of LUAD.

## 2 MATERIALS AND METHODS

### 2.1 The expression of SPANXC in normal tissues and LUAD patients

GTEx databases were used to assess testis‐specific expression of SPANXC at transcription level. The expression abundance (FPKM) of genes and transcripts was downloaded from the GTEx project (http://www.gtexportal.org/home/, dbGaP Accession phs000424.v6.p1). We obtained the expression of SPANXC in LUAD tissues by analysing RNA sequencing data of LUAD patients from TCGA projects (https://confluence.broadinstitute.org/display/GDAC/Home, 2014‐07‐13 release). The detailed characteristics of samples from GTEx and TCGA are shown in Table S1. Gene expression of SPANXC is dichotomized into above median and below median expression groups based on median cut‐off. Overall survival analysis curves were generated using Kaplan‐Meier method with Survival package in R. The survival difference between the dichotomized groups was evaluated using log‐rank test. DNA demethylation data of LUAD from TCGA were analysed with the R‐package.

### 2.2 RNA extraction, reverse transcription and the quantitative real‐time PCR

The total RNA was extracted from tissues or cultured cells with TRIzol reagent (Invitrogen), according to the manufacturer's protocol. One microgram of total RNA was reverse transcribed using PrimeScript RT Reagent Kit with gDNA Eraser (Takara). cDNA was used for subsequent qRT‐PCRs (SYBR, TaKaRa) according to the manufacturer's instructions. Results were normalized to the expression of GAPDH. The rest of primers are listed in Table S2.

### 2.3 Cell culture

Lung adenocarcinoma cell lines A549 and NCI‐H1299 were cultured in 1640 medium and DMEM (GIBCO‐BRL) supplemented with 10% foetal bovine serum (10% FBS), 100 U/mL penicillin and 100 mg/mL streptomycin in humidified air at 37°C with 5% CO_2_.

### 2.4 Transfection of cell lines

The siRNAs and plasmid were transfected into LUAD cells using Lipofectamine2000 (Invitrogen) according to the manufacturer's instructions. The sequences for siRNAs are listed in Table S1.

### 2.5 Cell proliferation assay

A count of 2000 cells per well was cultured in 96‐well plates (Corning Inc), and proliferation was evaluated by CCK8 for every 24 hours. For colony formation assay, a certain number of transfected cells were placed in each well of 6‐well plates and maintained in proper media containing 10% FBS for 2 weeks, replacing the medium every 4 days. Colonies were fixed with methanol and stained with 0.1% crystal violet (Sigma‐Aldrich) in PBS for 10 minutes. The colony formation was determined by counting the number of stained colonies. Triplicate wells were measured in each treatment group.

### 2.6 Cell migration and invasion assays

For cell migration and invasion assays, 24‐well transwell chambers with 8‐μm pore size polycarbonate membrane were used (Corning). Cells were seeded on the top side of the membrane pre‐coated with Matrigel (BD) (without matrigel for cell migration assay). Incubation for 24 hours, cells inside the upper chamber were removed with cotton swabs, while cells on the lower membrane surface were fixed and then stained with 0.5% crystal violet solution. Five fields were counted randomly in each well. All assays were performed in triplicate, and the experiment was repeated three times.

### 2.7 Co‐immunoprecipitation and mass spectrometry (LC‐MS/MS) assays

Co‐IP experiments were performed using a Pierce™ Classic Magnetic IP/Co‐IP Kit (Thermo) according to the manufacturer's instructions. Briefly, cell pellet was resuspended in Buffer A (10 mmol/L Hepes pH7.5, 1.5 mmol/L MgCl2, 10 mmol/L KCl, 0.5 mmol/L DTT and 1 mmol/L PMSF/Cocktail) for 10 minutes on ice, 0.25% NP‐40 was added for 5 minutes, and cytosol fraction and nuclear pellets were obtained by centrifugation at 13 000 RPM for 10 minutes. Nuclear pellet was then resuspended in Buffer C (20 mmol/L Hepes pH 7.5, 10% glycerol, 0.42 mol/L KCl, 4 mmol/L MgCl2, 0.2 mmol/L EDTA, 0.5 mmol/L DTT and 1 mmol/L PMSF/cocktail) 20 minutes on ice, and nuclear fraction was obtained after 13 000 RPM 10 minutes centrifugation. Cell fraction was mixed together, and 500 µg of lysate was used for one IP reaction. Antibodies was added, IP was performed on the rotating plate in 4°C for 3 hours, and 20 µL washed Magnetic beads was added and incubated for one hour. Quickly wash four times with Wash buffer (50 mmol/L TrisCl 7.9, 10% glycerol, 100 mmol/L KCl, 0.2 mmol/L EDTA, 5 mmol/L MgCl2, 10 mmol/L β‐ME 0.1% NP‐40). The expression plasmid of SPANXC was marked by flag. Antibody for Flag was from Sigma. The elutions of proteins were subjected to mass spectrometric analysis. LC**‐**MS/MS experiments were performed with a LTQ linear ion trap mass spectrometer (Thermo Finnigan) equipped with a microspray source.^16^

### 2.8 In vivo assay

NCI‐H1299 cells were stably transfected with overexpression of SPANXC and empty vector and harvested cells and washed with PBS, and resuspended at 2 × 10^7^ cells/mL. Then, the suspended cells were injected into the tail veins of nude mice, which were treated 60 days after injection. The presence of tumour nodules was determined and was counted. Our study was carried out in strict accordance with Guide for Care and Use of Laboratory Animals of the National Institutes of Health. Our project was approved by committee on the ethics of animal experiments of Nanjing Medical University.

### 2.9 Statistical analysis

All statistical analyses were performed using SPSS 20.0 software (IBM, SPSS). The significance of differences between groups was estimated by Student's *t* test, chi‐squared test or Wilcoxon test, as appropriate. Two‐sided *P*‐values were calculated, and a probability level of 0.05 was chosen for statistical significance.

## 3 RESULTS

### 3.1 CT gene SPANXC is activated by promoter hypomethylation and is associated with poor prognosis in LUAD

**Figure 1:**
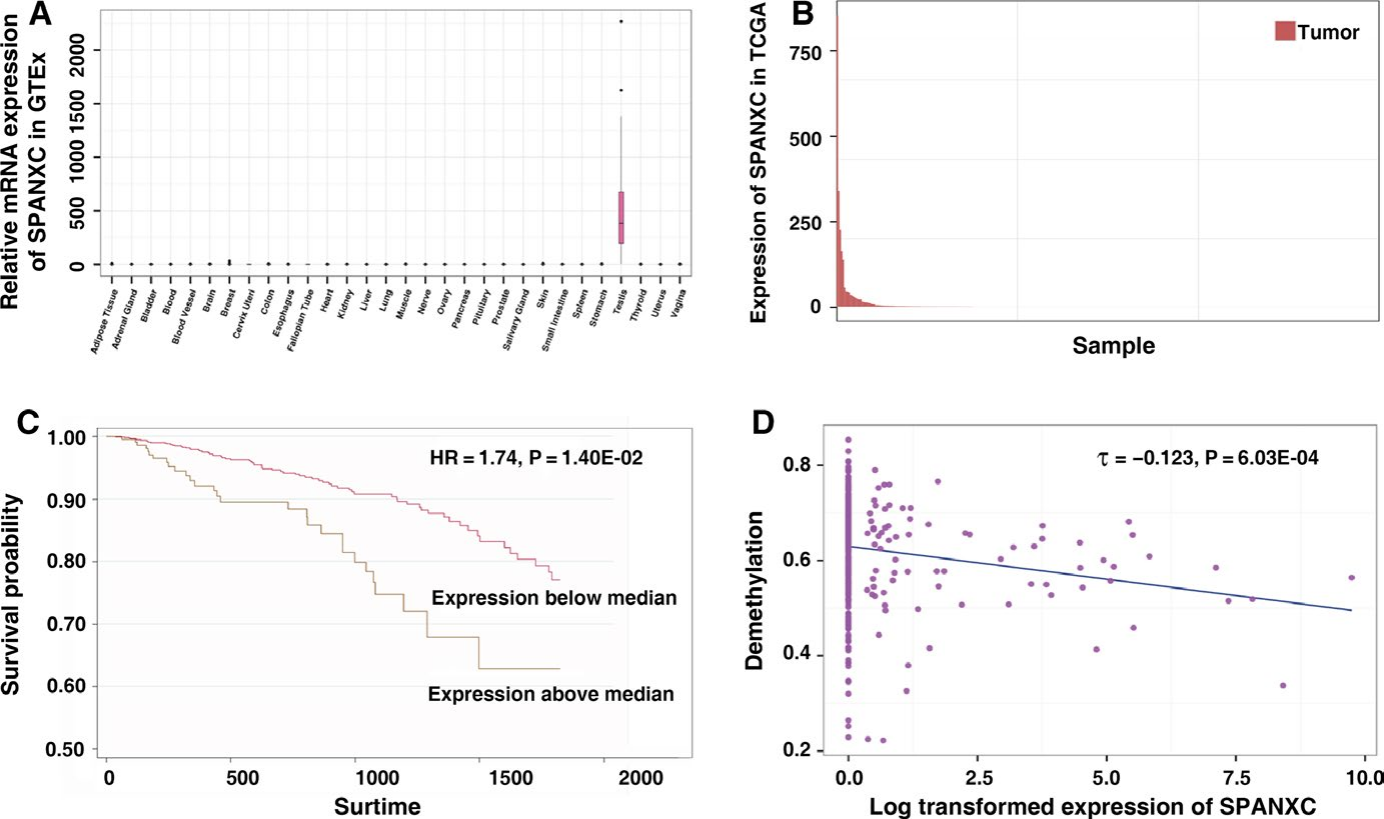
A, The mRNA expression levels revealed that SPANXC is restrictedly expressed in the testis. B, The TCGA project reveals that SPANXC mRNA is obviously activated in LUAD tissues. C, Kaplan‐Meier survival curve of LUAD patients’ overall survival based on TCGA database shows that patients with SPANXC activation have a reduced survival time than those without SPANXC non‐activation. D, The image shows the correlation between methylation and SPANXC expression in TCGA

Firstly, by utilizing transcription data from the GTEx, we found that SPANXC was a typical restricted expression in testis tissues (Figure 1A). Through integrating expression data of LUAD from the TCGA data, we found that SPANXC is frequently activated in LUAD patients (Figure 1B). These results indicated that SPANXC was CT gene and it was activated in LUAD.

Next, we inquired into the association between SPANXC activation and tumorigenesis of LUAD. Further analysis found that patients with higher SPANXC expression had a significantly poorer prognosis than those with lower SPANXC expression (HR = 1.74, *P* = 0.014, Figure 1C). Based on the methylation data of LUAD from TCGA, intriguingly, we found that the activation of SPANXC was due to the promoter hypomethylation of SPANXC (Figure 1D).

### 3.2 CT gene SPANXC regulates LUAD cell metastasis in vitro

**Figure 2:**
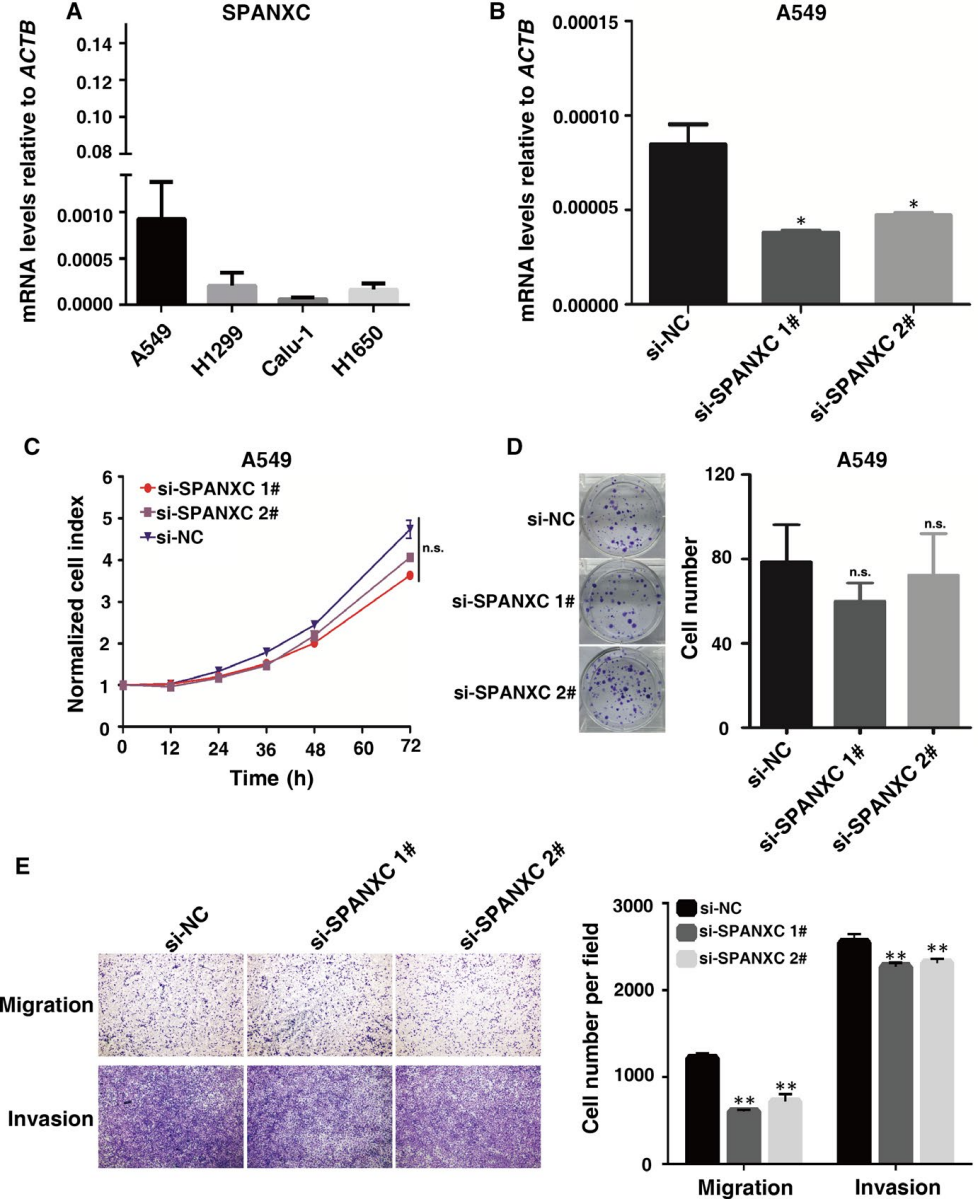
A, SPANXC relative expression was detected by RT‐qPCR in human LUAD cell lines. B, qRT‐PCR was performed to detect SPANXC expression after siRNA‐mediated knockdown. C, CCK8 assays were performed to determine cell proliferation of A549 cells after transfection. D, Colony formation assays of A549 cells after transfection. E, Transwell assays were used to investigate the migration and invasion abilities of NCI‐H1299 cells after transfection. *P < 0.05, **P < 0.01

**Figure 3:**
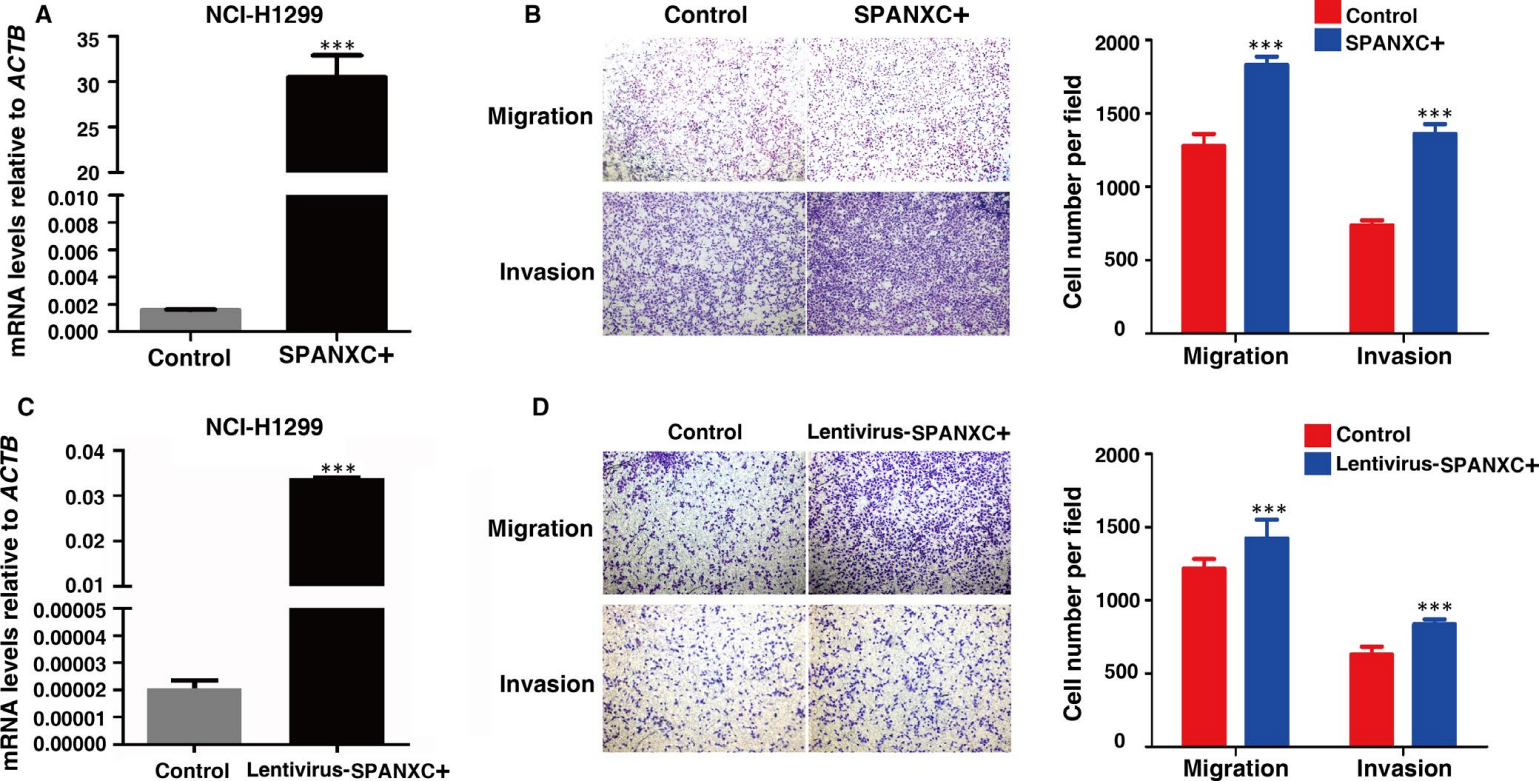
A, qRT‐PCR was performed to detect the SPANXC expression after plasmid‐mediated overexpression in NCI‐H1299 cells. B, Transwell assays were used to investigate the migration and invasion abilities of NCI‐H1299 cells after transfection of plasmid‐mediated overexpression. C, qRT‐PCR was performed to detect the SPANXC expression after lentivirus‐mediated overexpression in NCI‐H1299 cells. D, Transwell assays were used to investigate the migration and invasion abilities of NCI‐H1299 cells after transfection of lentivirus‐mediated overexpression. ***P < 0.01

To determine the role of SPANXC in tumorigenesis of LUAD, firstly, we determined the SPANXC expression levels in LUAD cell lines (Figure 2A). Then, we performed knockdown of SPANXC via siRNA in A549 cell lines which originally expressed relatively high SPANXC. After transfection with the specific siRNA (Figure 2B), there was no significant difference between treatment group and control group for cell proliferation and colony formation assay in A549 cells (Figure 2C and 2D). However, transwell assays found that knockdown of SPANXC resulted in significantly decreases in migration and invasion in A549 cells (Figure 2E). Conversely, overexpression of SPANXC can significantly promote cell migration and invasion in NCI‐H1299 cells (Figure 3A and 3B).

### 3.3 SPANXC promotes cell metastasis in vivo

**Figure 4:**
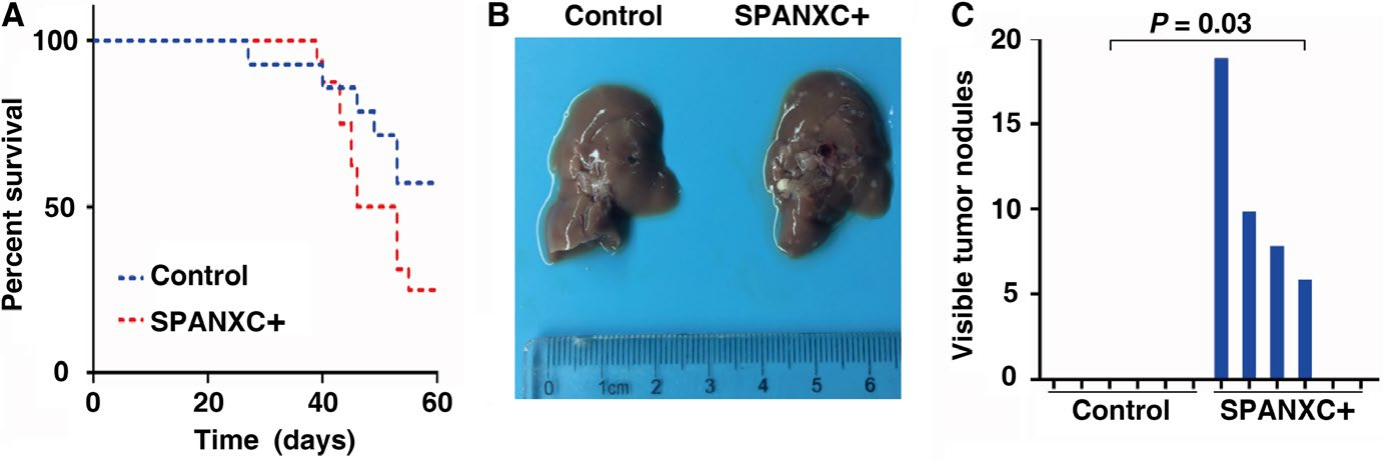
(A) The survival time of mice injected with NCI‐H1299‐SPANXC^+^ cells and the control group. B, and C, The number of metastatic nodules on the surface of the liver was significantly higher in mice injected with NCI‐H1299‐SPANXC^+^ cells than in mice injected with NCI‐H1299‐control cells

To further determine whether SPANXC affects cell metastasis in vivo, firstly, NCI‐H1299 cells stably transfected with SPANXC expression vector by lentivirus (Figure 3C). We found that lentivirus‐mediated stably transfected overexpression of SPANXC could promote cell migration and invasion in NCI‐H1299 (Figure 3D). Then, NCI‐H1299 cells with overexpression of SPANXC or control vector were injected into the tail veins of nude mice. Metastatic nodules on the surface of the liver were counted after 60 days. The mice injected with overexpression of SPANXC cells succumbed to death more rapidly than the control cells (Figure 4A). And the number of metastatic nodules on the surface of the liver was significantly higher than the control cells (Figure 4B and 4C). Taken together, SPANXC exhibits a pivotal role in cell metastasis of LUAD.

### 3.4 CT gene SPANXC could promote cell metastasis by binding to ROCK1

**Figure 5:**
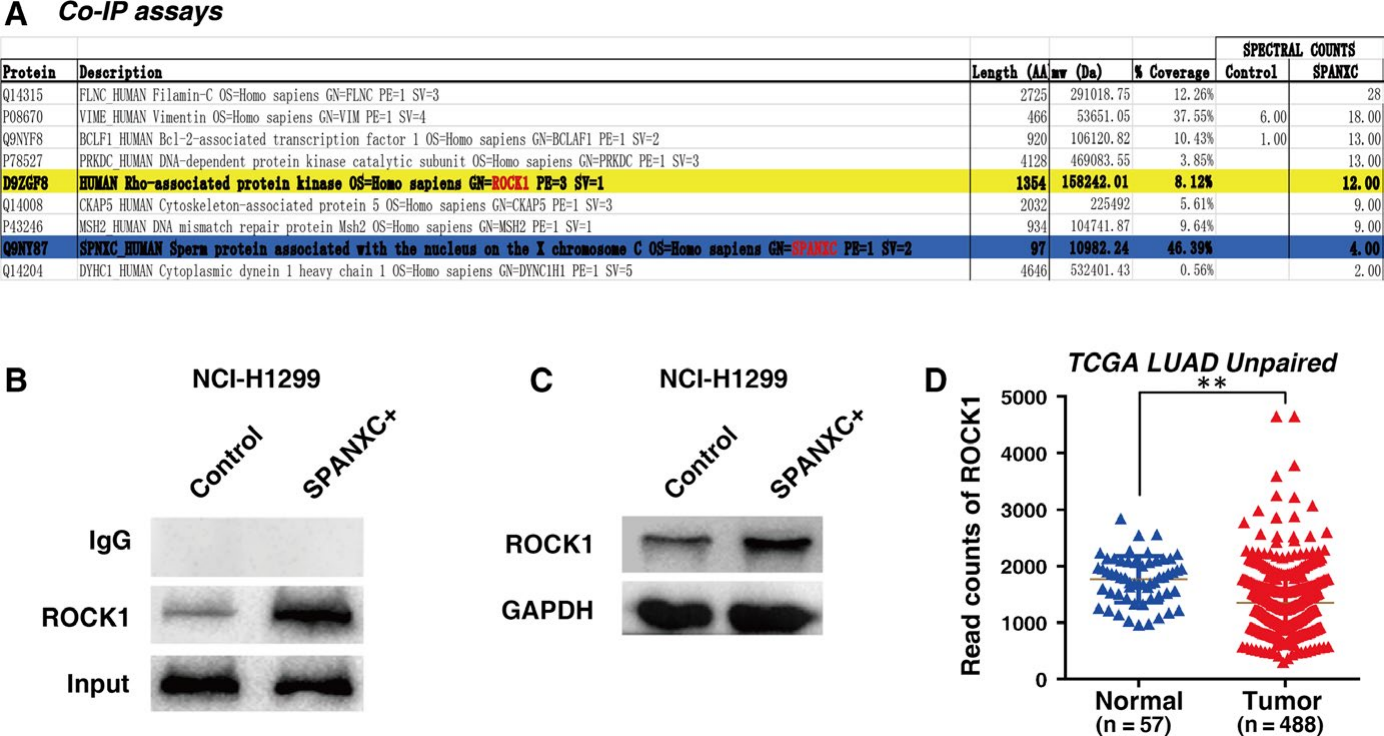
A, A list of differential protein potential SPANXC‐interacting protein candidates in NCI‐H1299 cells based on Co‐IP assays and mass spectrometry analysis. B, After overexpression of SPANXC in NCI‐H1299 cells, IP assays and WB assays were performed. SPANXC IP could retrieve ROCK1. C, Western blot assays detected the expression of ROCK1 after overexpression of SPANXC in NCI‐H1299 cells. D, The expression of ROCK1 in LUAD of TCGA data

Our data suggested that SPANXC may play an important role in cell metastasis of LUAD. To explore the mechanism for SPANXC in metastasis of LUAD, we performed co‐immunoprecipitation (IP) assays after overexpression of SPANXC, followed by mass spectrometry (MS), to assess the proteins interacting with SPANXC. The results showed that ROCK1 gene was obviously enriched after overexpression of SPANXC than that in control cells (Figure 5A). Then, we further validate the physical interaction between SPANXC and ROCK1 by Co‐IP and Western blotting assays (Figure 5B). Moreover, overexpression of SPANXC could indeed induce ROCK1 protein expression (Figure 5C). And we also found that ROCK1 has significant higher expression in LUAD samples after analysing from TCGA unpaired 488 LUAD tumour and 57 adjacent normal tissues (Figure 5D). ROCK1 has been participated in multiple cellular processes and pathologies, especially in metastasis process of tumorigenesis including LUAD.^17^, ^18^, ^19^ Our results indicated that SPANXC could promote cell metastasis by binding to ROCK1.

## 4 DISCUSSION

The procedure of germ cell formation and tumorigenesis has significant similarities, involving cell immortalization, migration and invasion, induction of meiosis.^7^ CT genes have been known as prospective cancer markers and objectives for therapy.^20^ The activation of CT gene expression in various types of tumours was observed.^21^, ^22^ Previous study found that the activation frequency of CT gene is much changeful depending on cancer type, the melanomas, the liver and lung cancers could show a high rate of recurrence.^21^, ^23^, ^24^ More and more studies showed that the activation of CT genes could affect on cells proliferation, apoptosis and metastasis of cancer cells, and also serve as a prognostic indicator.^25^, ^26^ For instance, significantly higher expression of CT gene MAGE‐A4 was detected in among squamous cell carcinoma of lung patients displayed obviously poorer survival than those without MAGE‐A4 expression.^27^

The SPANX genes could encode differentially testis‐specific proteins.^28^ SPANXC was a member of SPANX gene family that was located in Xq27.2.^28^, ^29^, ^30^ In our study, we found that SPANXC was a CT gene and was activated in patients of TCGA LUAD. The promoter hypomethylation was an important reason for activation of SPANXC in LUAD. Previous findings also indicated that demethylation is an important mechanism for CT gene reactivation.^9^, ^31^, ^32^ Then, functional assay in *vitro* and in *vivo* confirmed that SPANXC can contribute to the metastasis of LUAD. Mechanistic investigations found that SPANXC could bind to ROCK1 protein. Although it has been proved that SPANXC was a CT gene,^29^ the molecular mechanism of SPANXC was not clear. We observed that the interaction between SPANXC gene and ROCK1, ROCK1 was an important migration‐related gene in tumorigenesis.^17^ And ROCK1 also participated in epithelial‐to‐mesenchymal transition (EMT) in tumorigenesis.^33^, ^34^ Our results provided important evidence that SPANXC could promote the metastasis of LUAD cells, partly through interaction with ROCK1. Therefore, SPANXC may regulate cell metastasis by affecting EMT pathway. Our data further corroborated the resemblance between processes of germ cell development and tumorigenesis, including tumour metastasis.

In summary, CT gene SPANXC could be activated by promoter hypomethylation and indicated poor prognosis in LUAD patients. Moreover, SPANXC could regulate cell metastasis of LUAD both in *vitro* and in *vivo*. Our study demonstrated that CT gene SPANXC may be a candidate target for LUAD therapy.

## CONFLICT OF INTEREST

The authors declare that they have no competing interests.

## AUTHOR CONTRIBUTIONS

ZBH, EBZ and CW involved in conceptualization; WXW, CWY and QFQ involved in methodology; YC, SZC, YTC, SHJ, YDX and YYG performed data curation,; WXW, CZ wrote and prepared the original draft; EBZ involved in writing, reviewing and editing; ZBH involved in supervision.

### DATA AVAILABILITY

The datasets used and/or analysed during the current study are available from the corresponding author on reasonable request.
